# High efficacy of microbial larvicides for malaria vectors control in the city of Yaounde Cameroon following a cluster randomized trial

**DOI:** 10.1038/s41598-021-96362-z

**Published:** 2021-08-24

**Authors:** Christophe Antonio-Nkondjio, P. Doumbe-Belisse, L. Djamouko-Djonkam, C. S. Ngadjeu, A. Talipouo, E. Kopya, R. Bamou, M. P. Audrey Mayi, N. Sonhafouo-Chiana, D. L. Nkahe, R. Tabue, D. Achu Fosah, Jude D. Bigoga, P. Awono-Ambene, Charles S. Wondji

**Affiliations:** 1 Laboratoire de Recherche Sur Le Paludisme, Organisation de Coordination Pour La Lutte Contre Les Endémies en Afrique Centrale (OCEAC), P.O. Box 288, Yaoundé, Cameroun; 2 Faculty of Sciences, University of Buea, P.O. Box 63, Buea, Cameroon; 3 Faculty of Sciences, University of Yaoundé I, P.O. Box 337, Yaoundé, Cameroon; 4 Vector Borne Diseases Laboratory of the Biology and Applied Ecology Research Unit (VBID-URBEA), Department of Animal Biology, Faculty of Science of the University of Dschang, Dschang, Cameroon; 5 Department of Vector Biology, Liverpool School of Tropical Medicine Pembroke Place, Liverpool, L3 5QA UK; 6 Ministry of Public Health, National Malaria Control Programme, Yaoundé, Cameroon; 7 Laboratory for Vector Biology and Control, National Reference Unit for Vector Control, The Biotechnology Center, Nkolbisson-University of Yaounde I, P.O. Box 3851, Messa, Yaoundé, Cameroon; 8 Department of Biochemistry, Faculty of Science, University of Yaounde I, Yaoundé, Cameroon; 9 Centre for Research in Infectious Diseases, Yaoundé, Cameroon

**Keywords:** Ecology, Microbiology, Zoology, Diseases

## Abstract

The rapid expansion of insecticide resistance and outdoor malaria transmission are affecting the efficacy of current malaria control measures. In urban settings, where malaria transmission is focal and breeding habitats are few, fixed and findable, the addition of anti-larval control measures could be efficient for malaria vector control. But field evidences for this approach remains scarce. Here we provide findings of a randomized-control larviciding trial conducted in the city of Yaoundé that support the efficacy of this approach. A two arms random control trial design including 26 clusters of 2 to 4 km^2^ each (13 clusters in the intervention area and 13 in the non-intervention area) was used to assess larviciding efficacy. The microbial larvicide VectoMax combining *Bacillus thuringiensis* var *israelensis* (Bti) and *Bacillus sphaericus* in a single granule was applied every 2 weeks in all standing water collection points. The anopheline density collected using CDC light traps was used as the primary outcome, secondary outcomes included the entomological inoculation rate, breeding habitats with anopheline larvae, and larval density. Baseline entomological data collection was conducted for 17 months from March 2017 to July 2018 and the intervention lasted 26 months from September 2018 to November 2020. The intervention was associated with a reduction of 68% of adult anopheline biting density and of 79% of the entomological inoculation rate (OR 0.21; 95% CI 0.14–0.30, *P* < 0.0001). A reduction of 68.27% was recorded for indoor biting anophelines and 57.74% for outdoor biting anophelines. No impact on the composition of anopheline species was recorded. A reduction of over 35% of adult *Culex* biting densities was recorded. The study indicated high efficacy of larviciding for reducing malaria transmission intensity in the city of Yaoundé. Larviciding could be part of an integrated control approach for controlling malaria vectors and other mosquito species in the urban environment.

## Introduction

Africa’s population almost doubled during the last two decades, from about 665 million in 2000 to 1.1 billion in 2019^1^. This rapid demographic growth has resulted in a massive migration of the population from rural to urban areas. The rapid demographic changes in major sub-Saharan Africa cities which are also associated to large-scale unplanned urbanization including poor housing, poor drainage, inadequate waste management, multiplication of slums and environmental changes have significantly influenced the epidemiology of vector-borne diseases such as malaria and arboviruses and population health^2–5^. Malaria remains an important public health problem across the world affecting both rural and urban areas^6–9^. According to the latest world malaria report, 229 million malaria cases were reported in 2019^10^. Long-lasting insecticidal nets (LLINs) and indoor residual spraying (IRS) are considered as the cornerstone for malaria prevention^11^.

The large-scale deployment of these tools permitted to avoid about 1.5 billion malaria cases and 7.6 million malaria deaths between 2000 and 2019^12^. Roughly 1.9 billion LLINs have been distributed in sub-Saharan Africa between 2004 and 2019^10^. It is estimated that about 68% of households in sub-Saharan Africa had at least one LLIN in 2019 this suggesting a terrific increase compared to 5% in 2000^10^. However, control efforts are still affected by the rapid expansion of insecticide resistance. Almost all sub-Saharan countries have reported resistance to all four of the most commonly used insecticide classes^13,14^. Resistance to pyrethroids the compound used for impregnating bed nets is widespread^13,15,16^. Based on insecticide resistance monitoring data, many countries are now adopting new strategies to manage insecticide resistance and improve malaria control. These include switching to new control tools such as the deployment of pyrethroid-piperonyl butoxide (PBO) nets^17^ or the combination of different control tools or interventions^18,19^.

Larval source management (LSM) has proven in the past to be highly effective for lowering malaria transmission and even eliminating malaria vectors and disease transmission^20–22^. Historical literature reveals that the use of anti-larval mosquito control measures contributed to all successful eradication efforts^23–25^. Several studies reporting significant impact of larval control on malaria transmission or malaria morbidity have been registered across the continent^20,26,27^. However, despite historical facts and new evidences on larviciding efficacy this intervention is still not largely implemented for malaria control in sub-Saharan Africa due to the limited number of unbiased studies on its efficacy or effectiveness^27,28^. Systematic reviews produced in the recent years^27,29,30^, indicated low certainty evidences due to poor study design for many studies^27,30^. The World Health Organization (WHO) issued an interim position on larviciding recommending its use in moderate to low transmission settings as a supplement to core interventions (LLINs and IRS) in areas where aquatic habitats are few fixed and findable^11^. The intervention could be particularly indicated in urban settings or in highland areas where aquatic habitats are less important and malaria transmission moderate^31–33^. According to the WHO guidelines^34^, LSM could be integrated into malaria control or general mosquito abatement programmes once transmission has been reduced to low or moderate levels after the use of LLINs or IRS, or once these interventions have reached their maximum practical impact.

In Cameroon malaria remains an important public health problem. Between 2015 and 2018, the incidence of malaria cases increased across the country highlighting the need to intensify malaria control efforts^35,36^. Treated nets are the main measure implemented by the government to prevent malaria attacks. It is estimated that about 80% of households own a bed net and that close to 60% use treated nets regularly^36^. Apart from LLINs which were introduced in the country in the 1990s, there have been two pilot larval control trials initiated in the country to control *Culex quinquefasciatus* populations. The first one conducted in the 1990s in the city of Maroua which consisted of two treatments per year of all breeding habitats with *Bacillus sphaericus* as larvicide had a limited impact on the biting densities of *C. quinquefasciatus* mosquito populations^36^. The second pilot study conducted in Yaounde registered a 64% reduction of *C. quinquefasciatus* biting densities. However, because the authors did not included any control cluster the interpretation of their findings was limited^37^.

The city of Yaounde has a landscape with an alternation of both highland and lowland areas with over 90% of aquatic habitats located in lowland settings and could be an excellent environment to practice larviciding^38,39^. The population is approximately 3 million inhabitants and is characterised by a low malaria transmission pattern^40,41^. There have been so far not enough attempts to control malaria vectors using interventions suited to the landscape and ecological situation of the environment. We hypothesized that the implementation of larviciding in the city of Yaounde could result in over 40% reduction of mosquito biting densities and subsequently the entomological inoculation rate compare to sites where this intervention is not implemented. Generating evidences on the efficacy of larviciding in different epidemiological context could improve malaria control across Africa. In the course of the present study, a cluster randomised trial including 26 clusters of 2 to 4 km^2^ each divided into 2 groups 13 in the intervention area and 13 in the non-intervention area was conducted to assess the impact of larviciding on malaria transmission in the city of Yaoundé.

## Results

### Household characteristics

Baseline community and entomological surveys were conducted from February 2017 to July 2018. Some data deriving from these studies have been published previously^42–49^. Household characteristics were almost similar across the two study groups. Modern houses built up with cement (50% and 62.77%) and traditional houses constructed with mud, plank, and mix material (50% and 37.23%) were recorded. Most households (> 84%) owned at least a LLIN, 47% and 48% of households in the control and intervention area respectively had one LLIN for two people (Table 1). The majority of households had an average of 6 to 10 persons per household. Close to 20% of houses had screens on windows. The number of houses with ceilings was also similar between the two groups.

**Table 1:** House characteristics in the non-intervention and intervention areas.

Characteristics	Factors	Non-intervention areas		Intervention areas	
		N houses	%	N houses	%
Type of house	Modern	94	50.00	118	62.77
	Traditional	94	50.00	70	37.23
Occupants	[1–5]	72	39.13	78	43.33
	[6–10]	89	48.37	88	48.89
	≥ 11	23	12.50	14	7.78
Holes on walls	No	116	63.74	134	72.04
	Yes	66	36.26	52	27.96
Eaves	No	65	36.52	78	43.58
	Yes	113	63.48	101	56.42
Ceiling	No	124	67.03	102	55.74
	Yes	61	32.97	81	44.26
Screened windows	No	158	85.87	144	80.00
	Yes	26	14.13	36	20.00
At least one LLIN Per household	No	28	15.05	15	8.47
	Yes	158	84.95	162	91.53
Use of LLINs	No	24	13.04	15	8.47
	Yes	160	86.96	162	91.53
owning one LLIN for 2 people	No	99	52.38	98	51.65
	Yes	90	47.62	91	48.35
Vegetation close to the house	No	41	21.69	37	19.58
	Yes	148	78.31	152	80.42
Aquatic habitats close to the house	No	32	16.93	35	18.52
	Yes	157	83.07	154	81.48

N houses = Number of houses selected for interview and house characterisation; % percentage of houses, LLINs = Long Lasting Insecticidal Nets; Traditional house: houses with mud or plank walls (poorly constructed); Modern house: houses constructed with bricks or cement walls.

### Monthly distribution of anopheline larvae

Anopheline larval abundance was seasonal with high density during the short and long rainy seasons. The annual rainfall estimates was 761.4 mm in 2017, 845.4 mm in 2018, 3011.3 mm in 2019 and 2726.2 mm in 2020^50^. This pattern influenced breeding habitats availability and distribution in the city (Table 2).

**Table 2:** Distribution of anopheline and culicine larvae in breeding habitats at baseline and during the larviciding intervention.

	Baseline		Intervention		Percent reduction +
	Non-intervention area	Intervention area	Non-intervention area	Intervention area	
**Total of aquatic habitats**					
Checked	8313	8633	25,729	137,120	
Total number of water bodies with anopheline larvae (%) (95% CI)	1551 (18.66%) (17.74–19.61)	1150 (13.32%) (12.56–14.11)	1934 (7.52%) (7.18–7.85)	1102 (0.80%) (0.76–0.85)	85.46*
Total number with late instar anopheline larvae (%) (95% CI)	1096 (70.66%) (66.54–74.97)	772 (67.13%) (62.48–72.04)	1155 (59.72%) (56.33–63.27)	168 (15.24%) (13.03–17.73)	73.13*
Total number of water bodies with culicine larvae (%) (95% CI)	1528 (18.38%) (17.47–19.33)	1773 (20.54%) (19.59–21.52)	2523 (9.80%) (9.42–10.19)	5538 (4.03%) (3.93–4.14)	69.24*

+ Percent reduction = 100 − (Non LCI at baseline/LCI at baseline × LCI during intervention/non-LCI during intervention) × 100.

(Non-larviciding intervention area (Non LCI), Larvicidng Intervention area (LCI)): *P ≤ 0.001.

**Figure 1:**
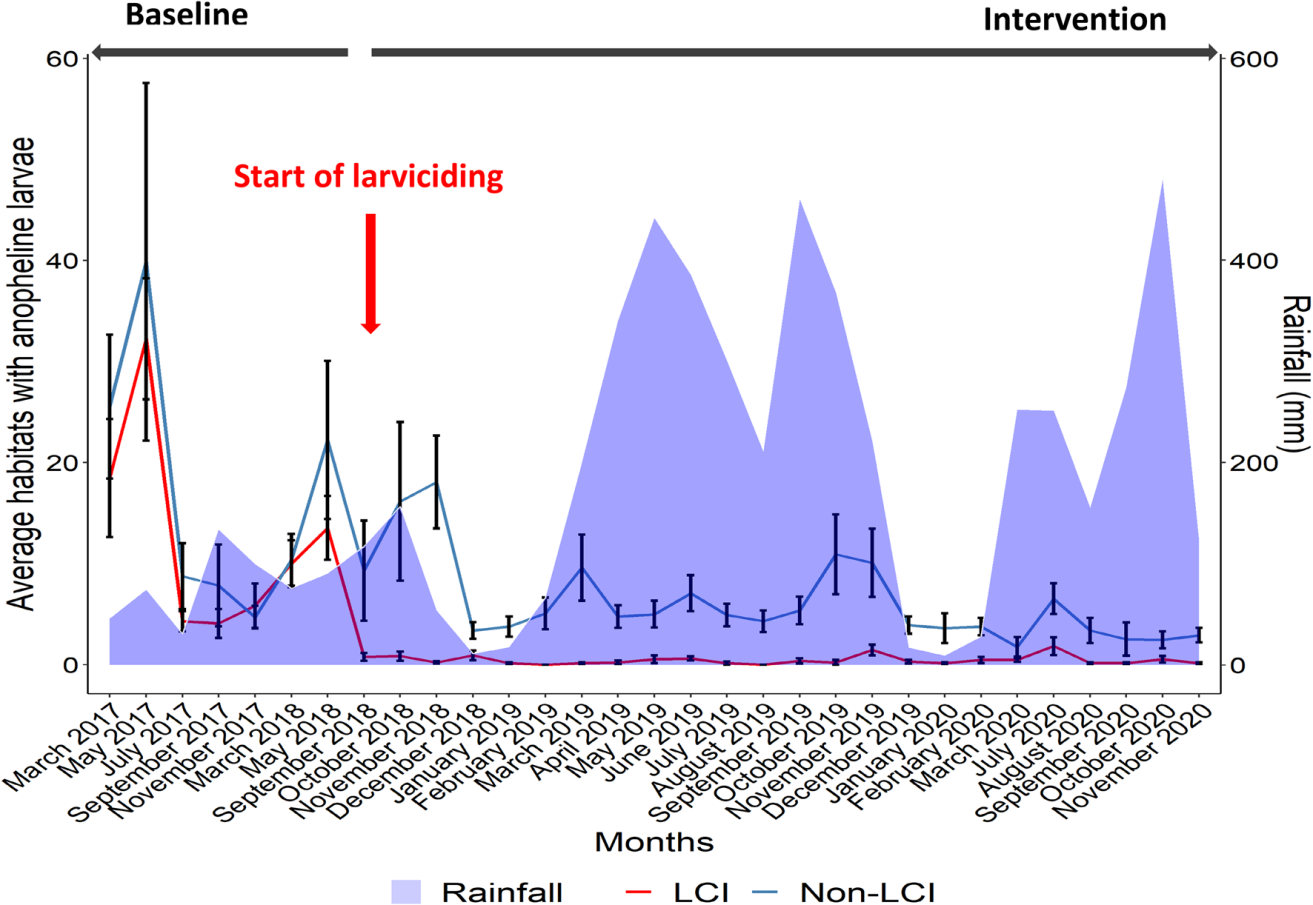
Distribution of aquatic habitats with anopheline larvae before and during the larviciding intervention ((Non-larviciding intervention area (Non LCI), Larvicidng Intervention area (LCI)).

The proportion of habitats found with early or late instar anopheles larvae at baseline was 13.32% (1150/8633) in intervention area and 18.66% (1551/8313) in non-intervention area. During the intervention period, only 0.80% of sites (1102/137,120) were found with anopheline larvae after larviciding treatments whereas, in non-intervention areas, 7.52% of sites (1934/25,729) were found with anopheline larvae. Taking into account the clustering by treatment group and by period, it appeared that larviciding treatment significantly reduced the chances of water bodies being colonised by anopheline larvae (OR = 0.15 95% CI = 0.07–0.32; *P* < 0.0001). The number of breeding habitats with late instar anopheline larvae was also reduced by over 73%. When was considered the effect of larviciding treatments on culicine larvae, a significant reduction of breeding habitats with culicine larvae could also be noticed (OR = 0.37 95% CI = 0.32–0.42; *P* < 0.0001). High fluctuation in the monthly distribution of breeding habitats with anopheline larvae closely associated with the rainfall pattern was recorded (Fig. 1).

### Changes in the presence of Anopheles larvae in aquatic habitats

In addition to unadjusted data analysis, a mixed linear modelling approach was used to better assess the impact of larviciding treatments. A total of 1131 measurements in both the intervention and non-intervention areas were taken into consideration for the modelling analysis. Results confirmed a significant reduction (*P* < 0.001) of breeding habitats with anopheline larvae in the intervention area compared to the non-intervention area, though a significant decline in the proportion of breeding habitats with *Anopheles* larvae for both non-intervention and intervention areas was generally observed with time (Table 3). Different factors including season, flooding, agricultural activities were found associated with a significant impact on the OR of the model (*P* < 0.005).

**Table 3:** Mixed effects logistic regression models of the impact of larviciding on the distribution of Anopheline larvae, and influence of other parameters during baseline and intervention periods.

Parameters	Baseline		Intervention	
	OR (95% CI)	*P* value	OR (95% CI)	*P* value
**Study group**				
Non-intervention	1			1
Intervention	1.17 (0.69, 1.97)	0.56	0.02 (0.01, 0.03)	< 0.0001
**Season**				
Dry	1			1
Rainy	Excluded		1.65 (1.44, 1.90)	< 0.0001
**Public works**				
No	1			1
Yes	Excluded		0.90 (0.58, 1.40)	0.63
**Flooding**				
No	1			1
Yes	0.37 (0.20, 0.67)	0.001	0.74 (0.61, 0.89)	0.001
**Agricultural acticities**				
No	1			1
Yes	0.71 (0.40, 1.25)	0.24	0.47 (0.28, 0.79)	0.004

Public works; refer to construction of roads or buildings, Agricultural activities: refer to the exploitation of land surface for the practice of agriculture; Flooding: refer to inundated areas. OR: odd ratio; 95% CI: 95% confidence interval.

The influence of physicochemical factors on anopheline larvae presence in aquatic habitats before and during the intervention could be found as Supplementary Table S1. Analysis conducted indicated low influence of these factors.

### Adult mosquito abundance

A total of 6664 anophelines were collected in the course of the study. Species collected included *An. gambiae s.l., An. funestus* and *An. ziemanni* (Table 4). A subsample of 2762 *An. gambiae* s.l., was processed by PCR and both *An. coluzzii* (88.42%) and *An. gambiae* (11.58%) were recorded. Within the *An. funestus* group, out of 299 mosquitoes processed, 280 (93.65%) were *An. funestus* s.s., and 19 (6.35%) were *An. leesoni*. In almost all districts *An. coluzzii* was the predominant species; followed by *An. gambiae*. No significant variation in the composition of *An. gambiae* and *An. coluzzii* before and during the intervention was recorded in both the intervention and non-intervention areas (*P* > 0.20) (The distribution of species before and during larviciding could be found in the Supplementary Figure S1). *An. funestus* was recorded in few sites and was particularly abundant in the site of Mendong located close to the periphery with large swamps.

**Table 4:** Composition of anopheline mosquito fauna in Yaounde.

	Pre-intervention		Intervention		Total (%)
	Non LCI N(%)	LCI N(%)	Non LCI N(%)	LCI N(%)	
*An. funestus s.l*	152 (7.10)	462 (18.65)	72 (4.75)	131 (24.72)	817 (12.26)
*An. gambiae s.l*	1976 (92.29)	1998 (80.66)	1422 (93.80)	392 (73.96)	5788 (86.8)
*An. ziemanni*	13 (0.61)	17 (0.69)	22 (1.45)	7 (1.32)	59 (0.89)
Total	2141 (34.98)	2477 (32.35)	1516 (24.21)	530 (8.46)	6664

Non-larviciding intervention area (Non LCI), Larviciding Intervention area (LCI).

**Figure 2:**
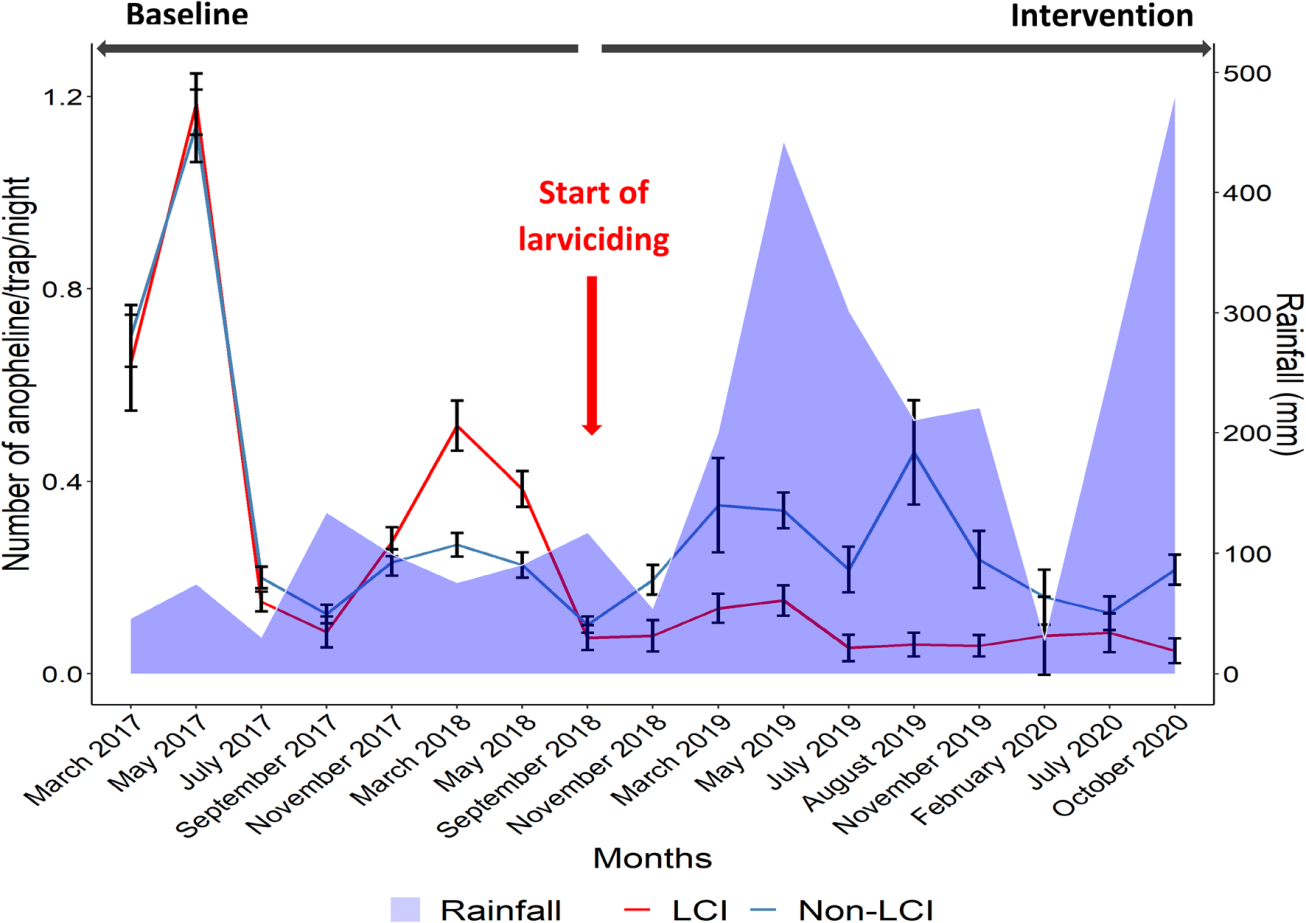
Fluctuation of anopheline biting density before and during larviciding intervention (Non-larviciding intervention area (Non LCI), Larvicidng Intervention area (LCI)).

Adult vector density was higher at baseline than for subsequent years throughout intervention in both the intervention and non-intervention areas (Fig. 2). After launching the intervention, a steady decrease in vector density was recorded in the intervention area. The average density of anopheline collected in the non-intervention clusters varied from 0.42 anopheline/trap/night at baseline to 0.23 anopheline/trap/night during the intervention. In the intervention clusters the average density of anopheline collected by CDC light traps varied from 0.47 anopheline/trap/night at baseline to 0.082 anopheline/trap/night during intervention. Larviciding was associated with 68% reduction of adult anopheline biting density. The density of mosquitoes collected indoor and outdoor in control and intervention area also varied significantly. The highest reduction was recorded with mosquitoes biting indoor 68.27% versus 57.74% outdoor (Table 5). When was compared the impact of the intervention on *An. gambiae* s.l., and *An. funestus* the two main vectors species in Yaounde, it appeared that *An. gambiae* s.l., biting density was reduced by over 71% whereas *An. funestus* density was reduced by 40% (Table 6).

**Table 5:** Crude entomological estimates of mosquito density and malaria transmission in Yaounde during the larviciding control trial.

Parameters	Baseline		Intervention		Percent reduction*
	Non-intervention area (95% CI)	Intervention area (95% CI)	Non-intervention area (95% CI)	Intervention area (95% CI)	
**Mean number of Anopheles per trap per night 95% CI**	0.42 (0.40–0.44)	0.47 (0.45–0.49)	0.23 (0.22–0.25)	0.082(0.075–0.09)	68.14
Indoor	0.62 (0.59–0.65)	0.67 (0.64–0.70)	0.35 (0.33–0.37)	0.12 (0.11–0.13)	68.27
Outdoor	0.12 (0.11–0.134)	0.12 (0.10–0.13)	0.071(0.06–0.08)	0.03 (0.02–0.03)	57.74
**Mean number of Culicine per trap per night 95% CI**	15.67 (15.56–15.77)	17.20 (17.09–17.31)	10.89 (10.82–10.98)	7.58 (7.51–7.64)	36.59
Indoor	20.82 (20.66–20.98)	22.04 (21.89–22.20)	15.7 (15.57–15.83)	10.76 (10.66–10.9)	35.26
Outdoor	7.91 (7.79–8.03)	8.40 (8.27–8.54)	4.33 (4.25–4.41)	2.65 (2.59–2.71)	42.37
**Average sporozoite rate** (%) Mean number of infected anopheles 95% CI	2.24 (1.60–3.00)	2.49 (1.86–3.25)	1.05 (0.57–1.76)	0.75 (0.20–1.93)	35.74
Indoor	2.45 (1.7–3.4)	2.52 (1.9–3.3)	1.10 (0.58–1.9)	0.65 (0.13–1.9)	42.55
Outdoor	0.88 (0.17–3.1)	2.11 (0.57–5.39)	0.67 (0.017–3.8)	1.47 (0.04–8.2)	8.49
**Annual entomological inoculation rate**^a^ Mean number of infectious bites per person per year 95% CI	5.50 (3.75–7.86)	6.83 (4.89–9.30)	1.41 (0.74–2.57)	0.36 (0.09–1.02)	79.53
Indoor	8.87 (5.97–12.76)	9.86 (6.97–13.63)	2.25 (1.13–4.05)	0.45 (0.08–1.44)	81.77
Outdoor	0.62 (0.068–2.48)	1.48 (0.34–4.09)	0.28 (0.01–1.78)	0.26 (0.01–1.63)	61.34

*Percent reduction = 100 − (Non LCI at baseline/LCI at baseline × LCI during intervention/non-LCI during intervention) × 100.

Non-larviciding intervention area (Non LCI), Larvicidng Intervention area (LCI).

^a^Estimated biting rate * 365 days per year * mean sporozoite prevalence * 1.6.

**Table 6:** Impact of larviciding treatments on biting *An. gambiae* s.l. and *An. funestus* densities in Yaounde.

Species	Baseline		Intervention		Percentage reduction^a^
	Non-intervention area (95% CI)	Intervention area (95% CI)	Non-intervention area (95% CI)	Intervention area (95% CI)	
*An. gambiae* s.l	0.39 (0.37–0.40)	0.38 (0.36–0.40)	0.22 (0.21–0.23)	0.061 (0.05–0.068)	71.54
*An. funestus* s.l	0.029 (0.025–0.034)	0.088 (0.08–0.096)	0.011 (0.009–0.014)	0.020 (0.017–0.024)	40.08

^a^Percent reduction = 100 − (Non LCI at baseline/LCI at baseline × LCI during intervention/non-LCI during intervention) × 100; Non-larviciding intervention area (Non LCI), Larvicidng Intervention area (LCI).

Crude analysis of sporozoites positive mosquitoes rate estimate showed a significant reduction of the sporozoite rate when binary logistic regression adjusting for years and group was applied (OR = 0.29; 95% CI = 0.10–0.80, *P* = 0.017). The entomological inoculation rate varied in the non-intervention area from 5.50 infected bites/person/year at the baseline to 1.41 infected bites/person/year during intervention. In the intervention area, the entomological inoculation rate dropped from 6.83 infected bites/person/year at the baseline to 0.36 infected bites/person/year during intervention. This accounted for 79% (OR 0.21; 95% CI 0.14–0.30, *P* < 0.0001) reduction of EIR.

Modeling the effect of larviciding intervention using general estimating equations after adjusting for clusters, years and seasons it appeared that at baseline, vector density and EIR in intervention and non-intervention area were readily comparable while the implementation of larviciding treatment significantly reduced the risk of being bitten by anopheline (*P* < 0.05), all this during the entire intervention period [beginning (2018), midway (2019) and end of the study (2020)] (Table 7).

**Table 7:** Impact of larviciding on biting anopheline densities and malaria transmission intensity estimated using generalized estimating equations (adjusting for years and periods).

Parameters	Estimated means (95% CI)	*P* value
**Baseline**		
Mar 2017–July 2018	0.1576 (− 0.08759, 0.4027)	0.1898
**Intervention**		
Sep–Dec 2018	0.09859 (0.05815, 0.1388)	0.0203
2019	0.08299 (0.05436, 0.1116)	0.0006
2020	0.06197 (0.009455, 0.1145)	0.0065
EIR (total)	0.0009444 (1.795e−05, 0.001871)	0.038

The influence of house characteristics on mosquito distribution in the intervention and non-intervention areas at baseline and during the intervention is presented in Supplementary Table S2. Parameters of the house (holes on the wall, absence of ceiling, absence of screens on windows) known to increase exposure to mosquito bites were found as contributing to a less important exposure risk in intervention area compared to non-intervention area.

### Evolution of *An. gambiae* s.l. susceptibility to pyrethroids and DDT

Studies conducted indicated a slight increase in the susceptibility status of *An. gambiae* females to both permethrin (mortality rates 34.16% at beginning to 39.26% at the end of the intervention) and deltamethrin (mortality rates 35.19% at beginning to 44.20% at the end of the intervention) during the trial (Table 8).

**Table 8:** Evolution of *An. gambiae* sl susceptibility to permethrin, deltamethrin and DDT during preintervention and intervention period in the city of Yaounde.

	Mortality rates/1014F allele frequency			
	Pre-intervention		Intervention	
	2017	2018	2019	2020
Permethrin 0.75%	34.16% (166/486)	7.71% (34/441)	2.07% (10/484)	39.26% (106/270)
Deltamethrin 0.05%	35.19% (411/1168)	12.30% (199/1618)	22.40% (97/433)	44.20% (80/181)
DDT 4%	1.33% (2/150)	3.05% (35/1146)	1.46% (5/342)	2% (4/200)
1014F Kdr allele frequency	60%	75%	67%	73%

The effect of larviciding with VectoMax G on non-target organisms could be found in supplementary Table S3. The study indicated no effect of larviciding treatments on non-target species diversity.

## Discussion

This study’s main objective was to assess the impact of larviciding on biting anopheline densities and malaria transmission intensity in the city of Yaounde. The present study used entomological outcomes as primary endpoint rather than epidemiological outcomes because of limited financial means. In Yaounde, over 90% of households own at least a net and over 70% of the population report using net regularly^43^. A high reduction of vector density and malaria transmission intensity was recorded with over 68% reduction of Anopheline densities collected using CDC-LT and 79% reduction of entomological inoculation rate. These figures are consistent with previous studies conducted across the continent supporting the high impact of antilarval measures on both entomological and epidemiological indicators^22,26,27,51^. The fact that a high number of clusters (including intervention and non-intervention areas) were used and monitored before and during the intervention, mosquito collection was undertaken using the Center for Disease Control Light trap (CDC LT) and the use of different teams involved in the treatment and the monitoring of field sites as recommended by WHO^21,52^, permitted to minimize the inclusion of bias (performance bias, selection bias, low sample size …) and further strengthen the quality of evidences deriving from the study. Well-conducted vector control field trials are essential to inform policy making and for evidence-based decision-making^52^. Important reduction of both indoor and outdoor biting anopheline densities was recorded confirming larviciding as a promising tool for controlling outdoor malaria transmission in urban settings.

During the study, continual application of larvicide was conducted rather than seasonal (during the rainy season) as done elsewhere^26^. This regular application of the larvicide led to a high reduction of breeding habitats with anopheline larvae, the density of anopheline larvae and late instar stages. These figures are consistent with previous findings^53,54^. Although studies conducted so far in Yaoundé suggested seasonal malaria transmission pattern^40,42,44^, it is possible that transmission could be occurring at an undetectable rate at some periods of the year due to the permanent presence of *An. gambiae* sl in the city and gametocyte carriers. This observation supports regular application of larvicide all year long at least during the first years of the intervention. Analysis of the landscape of the city of Yaounde and transmission risk pattern also indicated a heterogeneous malaria risk with some districts more affected than others^42,45^ and is in favor for emphasizing larviciding intervention in high risk zone. *An. funestus* was less intensely affected by the intervention compared to *An. gambiae* sl and could derive from the fact that *An. funestus* breed in water bodies covered by emerging vegetation which could reduce the quantity of larvicide granules getting to water surface and available for larvae whereas, *An. gambiae* s.l. is mainly found in water bodies without vegetation^55^. Limited impact of larviciding due to vegetation cover was reported in previous studies^56^.

Several physico-chemical parameters were monitored in the course of the study to assess their influence on mosquito distribution or larviciding treatments efficacy. Some of them including organophosphate, sulphate, conductivity and TDS were found to display different correlation patterns with larval density in intervention compared to non-intervention areas and could translate possible interaction with the larvicide. The possible influence of physico-chemical parameters on microbial larvicide efficacy deserves further assessment.

The composition of the anopheline fauna (particularly *An. gambiae* and *An. coluzzii*) did not changed significantly in the intervention and non-intervention areas before and during the intervention, which could suggest similar susceptibility status to larvicide of the two species as earlier suggested for insecticides^57^. Yet, studies conducted so far also indicated different insecticide resistance mechanisms in both *An. gambiae* and *An. coluzzii* in the city of Yaounde^47^. Insecticide resistance is largely spread across Yaounde^58–60^ but this seems to have had no impact on the effectiveness of larviciding treatments, since high reduction in anopheline density was recorded. A recent study in the city of Yaoundé indicated longer larval development time for resistant mosquitoes compare to susceptible^49^. This specific characteristic could increase the exposure of resistant mosquitoes to larvicide and increase mortality rate among insecticide resistant larvae. The following further supports the additional benefit of larviciding which could act as a complementary tool for insecticide resistance management^61^. Anti-larval measures could induce a reversal of resistance to pyrethroids and extend the efficacy of pyrethroid LLINs^62^. Microbial larvicides are also known to be highly efficient, specific and safe to use^21^. Moreover, the risk that resistance could emerge is very low due to the complex mode of action of these larvicides particularly *Bacillus thuringiensis* which has up to four different endotoxins^21^. Following up the susceptibility profile of anopheline mosquitoes suggested no significant evolution of pyrethroid resistance and kdr alleles. However at this stage, it is not clear whether this pattern could be associated to the implementation of larviciding activities or reflect seasonal or temporal variations in Yaounde.

A moderate reduction of adult *Culex* species biting density was recorded. The limited impact of larviciding treatments on this species could be due to the fact that these mosquitoes breed in different types of habitats such as pit latrines, which were not specifically targeted during larviciding treatments. It may also be possible that the impact of larviciding treatments in drains which are also preferential breeding habitats for *Culex* could have been limited due to the presence of solid wastes and many hiding places which could have limited the distribution of larvicide in the water^45^. *Culex* mosquitoes in Yaounde have also been reported to display a high insecticide resistance profile^46,63^.

As for houses, various factors allow mosquitoes to easily get in, including holes in walls, presence of opened eaves or absence of ceiling, which were proven to have a limited influence on indoor biting mosquito’s density during intervention, compared to the baseline period in intervention areas. Also, factors preventing mosquitoes from entering houses, such as presence of screens on windows or use of LLINs were found to induce better protection in areas where larviciding intervention was implemented compared to non-intervention areas. Better housing has always been regarded as a factor that could improve protection against mosquito bites in urban settings^48,64–66^.

The impact of the use of the microbial larvicide VectoMax on non-target organisms was also monitored and no significant impact on the non-target microfauna (*Cladocerans, Rotifers, Ostracods* and *Copepods*) was recorded. A high diversity of the microfauna was instead recorded in intervention areas. Further studies are needed to assess the effect of this larvicide on the aquatic macrofauna.

This study had some limitations. (1) Due to limited financial resources, the study mainly focused on entomological outcomes as primary endpoints rather than epidemiological outcomes as generally done. However, it provided a proof of concept that larviciding could be a suitable measure for reducing malaria transmission intensity in Yaounde. (2) The study relied on self-report assessment to measure LLIN coverage and use. This could have biased the interpretation of the added effect of larviciding on LLINs. (3) The study did not assess the cost-effectiveness of larviciding which is very important for policymakers.

## Conclusion

This study sets out to advocate the fact that the use of larviciding as a complement to LLINs could be a viable solution for controlling malaria transmission in Yaounde, in a context of rapid expansion of insecticide resistance across Africa and outdoor malaria transmission. The study provided strong evidence supporting the use of larviciding as a main intervention in urban settings. Results obtained should be considered by national control programmes and local Government to implement tailored control approaches to improve the fight against vector-borne diseases in urban settings. Further studies should be carried out to assess the impact of larviciding on epidemiological outcomes in Yaounde, the cost-effectiveness of larviciding with microbial larvicide and ways to involve community in vector control activities to ensure the sustainability of such interventions.

## Materials and methods

### Study area

**Figure 3:**
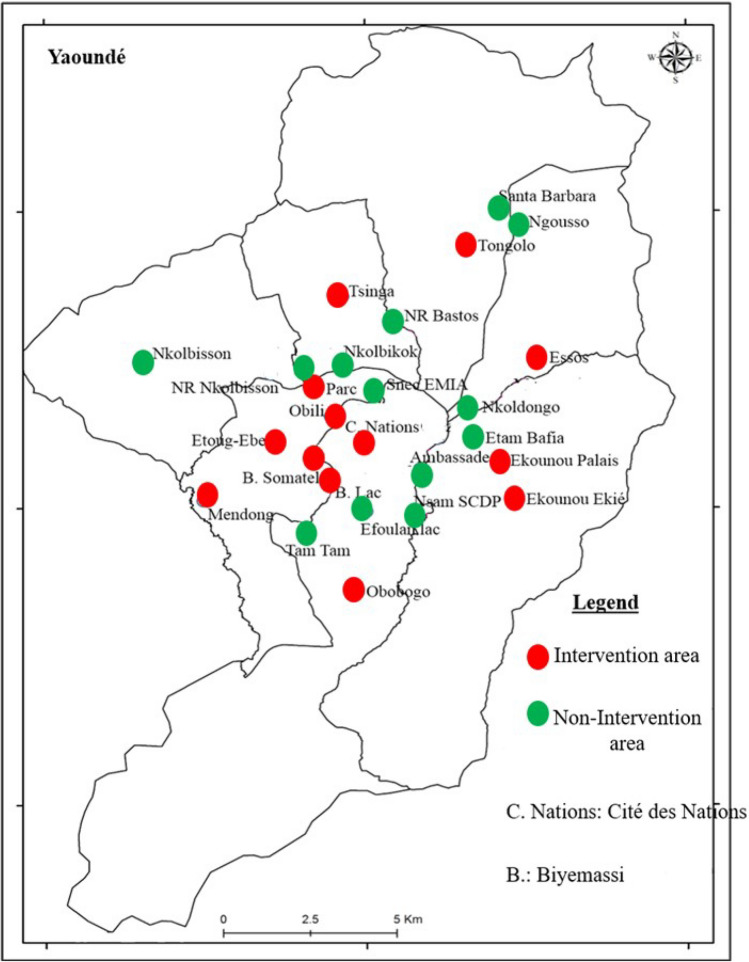
A map of the city of Yaoundé presenting intervention and non-intervention sites. The administrative division of Cameroon is available in open access on the OpenStreetMap platform (https://www.openstreetmap.org/search?query=cameroon#map=6/ 7.406/12.283). ArcGIS version 10.2.2 sofware (ESRI, Redland, CA, USA; https://www.esri.com/enus/arcgis/about-arcgis/overview) was used to generate the map showing study sites in Yaoundé.

The study was conducted in Yaoundé the capital of Cameroon (3° 52′ 12 N; 11° 31′ 12 E) (Fig. 3). Yaounde is located 726 m above sea level and receives up to 1700 mm of rainfall annually. It displays an equatorial climate with two rainy seasons extending from March to June and from September to November lasting 7 to 8 months. Despite its geographical location in the equatorial forest domain, the extension of settlements has significantly reduced the forest cover mainly found in nearby rural areas. The city extends 20 km wide and about 25 km long. Yaounde landscape comprises highlands and lowlands areas crossed by several rivers. Lowland areas are exploited during the dry season for agriculture. Houses are built on both hill slopes and in lowlands. Main rivers crossing the city include rivers Mfoundi, Ekozoa, Biyeme and Mefou.

### Sample size justification

Assuming a baseline density of 1 anopheline/CDC-trap/night k = 0.3 with collection conducted in 10 houses/cluster using 13 clusters/arm, it was estimated that the study will have over 80% power to detect up to 33% reduction of the density of anopheline collected using CDC-LT at the 5% significance level.

### Study design and larviciding activities

**Figure 4:**
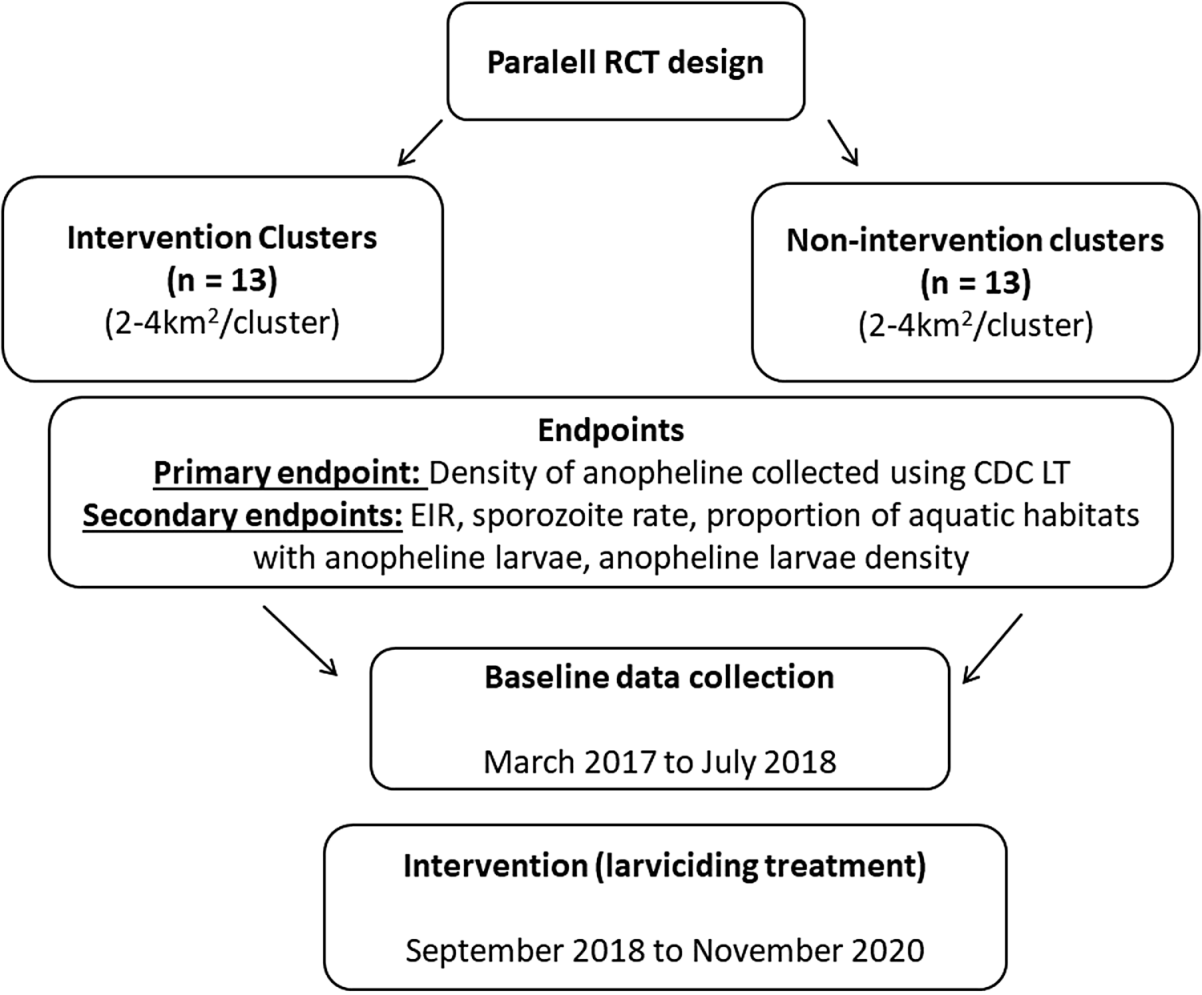
Trial profile design (RCT: Random Control Trial).

The primary objective of the trial was to assess the effect of larviciding on anopheline mosquito densities and malaria transmission rate in Yaounde. A parallel cluster randomised trial was conducted in twenty-six districts referred to as clusters (Fig. 4). Thirteen clusters served as control whereas the thirteen remaining were the intervention areas. Each cluster was an area of 2 to 4 km^2^ crossed by a river system encompassing both lowland and highland areas. The lowland part for the majority of clusters was sparsed and exploited for agriculture or with human constructions. The evaluation zone was situated at the center of each cluster always in the lowland area. Clusters were separated from one another by a distance of 500 m to 1 km to minimize mosquito spillover from non-intervention to intervention sites. Administrative boundaries (including roads, railways) were used to determine the limit of clusters. Baseline entomological data were collected from all clusters for 17 months, from March 2017 to July 2018. After this period microbial larvicide was applied in 13 clusters for 27 months (September 2018 to November 2020) (Fig. 4). Adult biting densities collected using CDC light traps were used as the primary outcome. At the baseline, it was noticed that > 90% of households owned at least one LLIN, but only 58.5% had one LLIN for two people as requested by the WHO^36^. At the end of the baseline sampling period, all clusters were ranked according to adult anopheline biting density. Clusters with similar biting density were grouped into pairs and from each pair, one cluster was randomly selected as the intervention site and the other as control using a computer-assisted programme.

### Intervention

**Figure 5:**
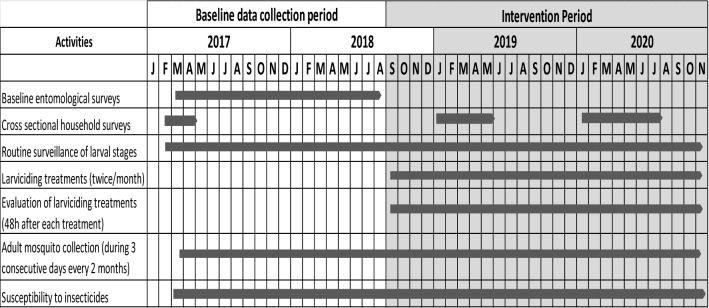
Duration of activities planned during the larviciding trial in the city of Yaounde.

In intervention clusters, all aquatic habitats which could serve as potential breeding sites for mosquitoes were treated from September 2018 to November 2020 (Fig. 5). It was assumed that when larvicide was applied to the entire cluster, the buffer zone and the fact that the evaluation was conducted at the centre of the cluster, could reduce mosquito spillover from non-intervention sites to intervention areas. Treatments were conducted using the larvicide VectoMax (Valent Biosciences Corporation, USA) a granule formulation (CG) containing as active ingredients both *B. thuringiensis israelensis* (Bti), strain AM65-52 (45 g/kg) and *B. sphaericus* (Bsph) strain ABTS-1743 (27 g/kg). VectoMax contains 50Bs international toxic units per mg of the product. According to WHO recommendations^67^ this larvicide should be used at the dosage of 500 to 1500 mg/m^2^ in open water bodies (pools, temporary puddles and artificial containers) with an effect lasting for 2 to 3 weeks. During the trial aquatic habitats were treated once every two weeks by hand application of the larvicide. Field applicators were recruited from local communities. They were supervised during each field trip by one field supervisor in each zone and trained for one month before starting larviciding activities. Application of larvicide was conducted early in the morning between 7 and 11 AM to avoid the hottest time of the day. Teams of three to four male adult applicators conducted the application of larvicide across each cluster. A minimum of 200 aquatic habitats/cluster were treated per day by a team (there were more breeding habitats in the rainy season compare to the dry season). Each team performing larviciding treatments used about 15 to 50 kg of larvicide/cluster/day depending on the size of the cluster and the availability of aquatic habitats.

### Endpoints

To assess the impact of the LSM intervention we used as primary outcome adult anopheline biting density collected using CDC light traps. Secondary outcomes included the entomological inoculation rate, the sporozoite rate, the presence of anopheline larvae in breeding habitats and larval density.

### Larval vector abundance

During the study, all aquatic habitats (with or without larvae) were identified and characterised. Their size, physico-chemical characteristics and the presence or absence of anopheline and culicine larvae were recorded every month.

During the intervention, water collection points were checked every week in the intervention area to find out the number of habitats containing early and late instar larvae, to determine the effectiveness of larvicide application. Surveillance of treated aquatic habitats was conducted 48 h after the treatment by a team of two people (different from those who undertook the treatment) who visited at least 50% of the treated area and all breeding habitats found with larvae were retreated. Checking larvae in aquatic habitats was also conducted in non-intervention sites once every month to capture the progression of mosquitoes in these sites. All water bodies encountered were geo-located using a Garmin eTrex GPS and recorded in a GIS database for analysis.

Water bodies were analysed to check the presence of mosquito larvae. Aquatic habitats with or without anopheline larvae were all described and registered. The immature stages of mosquitoes were collected using standard dipping technique^68^. Using a 350 ml deeper, three to five dips were performed for small aquatic habitats of less than 1 m^2^; and five to ten dips for aquatic habitats of more than 1 m^2^. For some habitats such as tyres or footprints which could be too shallow during certain periods, larval collection was conducted using a pipette. The average larval density (N) was estimated by calculating the ratio of the number of larvae collected per dip (using a dipper with a volume of 350 ml). Once collected larvae were classified according to their stages: early instars larvae (L1 and L2), late instars (L3, L4) and pupae. Anopheline larvae were separated from the culicines using morphologically identification keys^69,70^. Each anopheline larvae specimen was stored individually at − 20 °C and molecularly identified.

### Physico-chemical characterization of aquatic habitats

Parameters recorded in each breeding site included habitats type, size, depth, exposure to sunlight, presence/absence of vegetation, distance between each water point and the nearest human dwellings, the presence/absence of predators, organic pollution status, proportion of water surface covered by vegetation or algae, aquatic habitats type (stagnant water pools, gutters, well, tire print, footprint, pit latrine….). In addition to these, the following parameters were also recorded: Total Dissolved Solids (TDS), pH, temperature, conductivity using a Jenway multiparametric probe. The concentrations of sulphates, organophosphates, hydrogen peroxide (H_2_O_2_), turbidity, iron and calcium were analyzed using a Wagtech spectrophotometer^71^.

### Adult mosquitoes sampling

Adult mosquitoes were sampled using the Centre for Disease Control and Prevention Light Traps (CDC-LTs) both indoor and outdoor. Collections were performed once every two months from March 2017 to November 2020. CDC-LTs were placed indoor and outdoor in 10 homes per district. Houses used for adult mosquito collection were selected randomly. Every 50 to 100 m one house was selected randomly. If consent was not obtained from house owners the house situated next was chosen. The same houses were used for mosquito collection throughout the study. Mosquito collection were undertaken at the center of each cluster. Collections were undertaken from 7 pm to 6 am during 3 consecutive days per district per month in each district.

### Mosquito processing

Once collected, anophelines were separated from culicines using morphological identification keys of Edwards et al.^72^. Anopheline species were identified using morphological identification keys of Gillies and De Meillon^73^ and Gillies and Coetzee^74^. Mosquitoes belonging to the *Anopheles gambiae* complex were further processed by PCR^75^ to identify between *An. coluzzii* and *An. gambiae* the two members of the complex found in Yaoundé. Molecular identification of members of *Anopheles funestus* group was conducted according to Koekemoer et al.^76^. Both adult and larvae were molecularly identified. DNA extracted from wings and legs or the whole larvae according to Livak method^77^ was used for analysis. Each anopheline specimen was stored individually in a numbered Eppendorf tube containing desiccant, archived and kept in the freezer at − 20 °C. Heads and thoraxes of female anophelines were tested to check the presence of circumsporozoite protein (CSP) of *Plasmodium falciparum* by ELISA, as described by Wirtz et al.^78^ or using TaqMan method^79^.

### Insecticide bioassay

Adult females *An. gambiae* s.l. reared from larval collections in different collection sites were tested using three insecticides (deltamethrin 0.05%, permethrin 0.75% and DDT 4%) following WHO guidelines^80^. *An. gambiae* s.l. females aged 3–4 days reared from larvae collected on site were placed in batches of 20 to 25 mosquitoes per tube. The mosquitoes were then transferred into tubes with insecticide-impregnated papers and exposed for 1 h. The insecticide susceptible strains *An. gambiae* s.l. Kisumu and Ngousso strains were used as control to assess the quality of impregnated papers. The number of mosquitoes knocked down by the insecticide was recorded after 1 h exposure; then, mosquitoes were fed with a 10% sugar solution and the number of dead mosquitoes recorded 24 h post-exposure. Mosquitoes subjected to untreated papers were systematically included as controls. To detect the presence of the *kdr* alleles (L1014F and L1014S) conferring resistance to DDT and pyrethroids, DNA extracted from a sub-sample of *An. gambiae* s.l. females were screened using the TaqMan assay^81^.

### Household surveys

Household surveys were conducted using a questionnaire. The following information was recorded: house characteristics (building material), geographical coordinates of the house, features to prevent mosquitoes from entering (screen, ceiling, close eaves) or those allowing mosquito to enter (holes on the wall, absence of ceiling, open eaves), presence and usage of LLINs, or other antimalarial measures, socio-demographic information of each household (occupation, education level, number of inhabitants per house).

### Blinding

Entomological data collection was not blinded to the assignment of mosquito larval control interventions in the different clusters. Field applicators were blinded to the sites selected for larval surveys. Residents were aware of the implementation of the intervention. Adult mosquito collection was conducted using CDC light traps to avoid performance bias. Collections were conducted each month for three consecutive days to lessen variation due to rainfall or temperature. Laboratory technicians processing samples or conducting laboratory analysis were blinded to the identity of the cluster.

### Ethical clearance and authorizations

The study was conducted under the ethical clearance N° 2016/11/832/CE/CNERSH/SP delivered by Cameroon National Ethics Committee on Human Health. Further informed consent was obtained from the senior division administrator of the city of Yaoundé and each local District Medical Officer. Verbal and written informed consents were obtained from all respondents and the study purpose was explained to them. Permission to carry the trial was given by the Ministry of Public Health of Cameroon (Reference: 631-06-17). All experiments were performed in accordance with relevant guidelines and regulations.

Research and import permit for the use of VectoMax in Cameroon was granted by the Minister of Trade (Reference IF014167; IF021096; IF031126).

### Data analysis

Data were collected on forms, checked first to ensure they were filled comprehensively, then recorded in excel databases. Linear mixed models with random intercepts and Generalized Estimating Equations were used to assess the effect of larviciding treatment on the presence of anopheline larvae (early and late instars) in water collection points as well as adult anopheline density, infection rate and Entomological Inoculation Rate **(**EIR) respectively. In a preliminary analysis, follow-up curves for the non-intervention and intervention areas were constructed to visualize differences in the responses between the two sites. Average trends and local polynomial regressions of the presence of anopheline larvae (early and late instars) in water collections, *anopheline* density, infection rate and EIR with date of evaluation were also constructed separately for the different groups to further visualize these differences. We also estimated a null model with random intercept and calculated the intraclass correlation coefficient (ICC) associated with the presence of anopheline larvae, *anopheline* density, infection rate and EIR respectively. Generalized Estimating Equations were further used to assess the impact of larviciding on anopheline larvae presence in aquatic habitats with clustering by water bodies and zone included as random effects. GEE analyses were also used to assess the impact of larviciding on adult *Anopheline* density, infection rate and EIR by treating larviciding as a categorical independent factor in the model. Comparisons were adjusted for survey periods (months), years, baseline densities and clustering by traps and cluster. In all these cases, the identity link function with a Gaussian distribution was used, and we resorted to model with independent correlation structures. Clusters were treated as the geographic location, year as the indicator of time, larval presence in aquatic habitats, anopheline densities and EIR estimated as means for each cluster over the full year or the duration of the intervention. Although in the present analysis Clusters were used as the experimental units for the analysis which allowed the impact of larviciding to be estimated, some individual factors operating at the house level were also assessed. We control for individual level factors such as houses by treating individual houses as experimental units and preventing cluster larviciding covariance by restricting our analysis to the 466 houses used for mosquito collection surveyed during both the baseline and intervention period. A first order autoregressive relationship was applied for all repeated measurements. All analyses were carried out with the R 4.0.2 software using the R packages nlme, ggplot2, plyr, lattice, car, effects, emmeans and data.table. Odds ratios and risk ratio were calculated and adjusted for the year of intervention, cluster and season. Binary logistic regression was used to assess the distribution between species and physicochemical parameters in intervention and non-intervention areas. The Entomological inoculation rate was calculated by multiplying the mean density of mosquitoes collected in light traps in each cluster by the proportion of infected mosquitoes, by the number of days in the year and by 1.6 (the coefficient of underestimation of light trap compare to human landing catches after preliminary studies). The percentage reduction of mosquito densities and EIR following larviciding intervention were estimated using Mulla formula^82^.

## Supplementary Information

Supplementary Information.

## Data Availability

The datasets supporting the findings of this paper are included in this paper.
