# Supplementary material for: High efficacy of microbial larvicides for malaria vectors control in the city of Yaounde Cameroon following a cluster randomized trial
Source: Sci Rep. 2021 Aug 24;11:17101. doi: 10.1038/s41598-021-96362-z (PMC8385066; doi:10.1038/s41598-021-96362-z)
Supplement: Supplementary file 1 — Supplementary Information. [file 41598_2021_96362_MOESM1_ESM.docx]

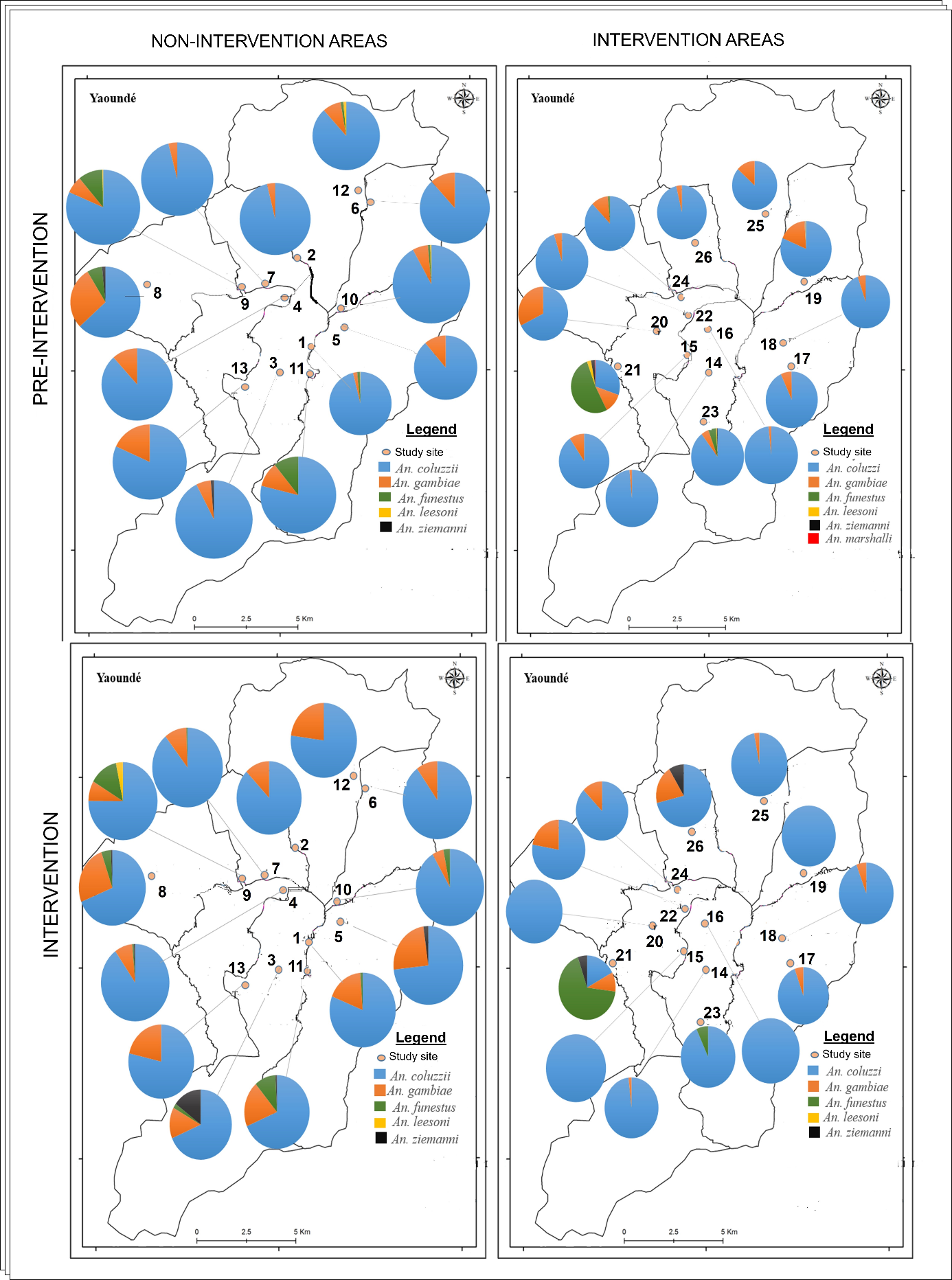


**Supplementary Figure S1:** Distribution of anopheline species in Yaounde before and during the intervention

**Legend :** 1 : Mvolyé ; 2 : Bastos nouvelle route ; 3 : Efoulan ; 4 : Emia ; 5 : Etam-bafia ; 6 : Ngousso ; 7 : Nkolbikok ; 8 : Nkolbisson ; 9 : Nkolbisson nouvelle route ; 10 : Nkolndongo ; 11 : Nsam ; 12 : Santa-Barbara ; 13 : Tam-Tam; 14 : Biyemassi lac ; 15 : Biyemassi Somatel ; 16 : Cité des nations ; 17 : Ekounou-Ekié ; 18 : Ekounou-Palais ; 19 : Essos ; 20 :Etoug-Ebe ; 21 : Mendong ; 22 : Obili ; 23 : Obobogo ; 24 : Parc Labogenie ; 25 : Tongolo ; 26 : Tsinga.

(The administrative division of Cameroon is available in open access on the OpenStreetMap platform (https://www.openstreetmap.org/search?query=cameroon#map=6/ 7.406/12.283). ArcGIS version 10.2.2 sofware (ESRI, Redland, CA, USA; https://www.esri.com/enus/arcgis/about-arcgis/overview) was used to generate the map showing study sites in Yaoundé).

SupplementaryTable S1: Association between physico-chemical parameters and anopheline larvae density in aquatic habitats at baseline and during larviciding treatment in non-intervention and intervention areas

| **Baseline** | Non-intervention areas | | | |  | Intervention areas | | | |
| --- | --- | --- | --- | --- | --- | --- | --- | --- | --- |
| Parameters | N | Means ± SE | R^2^ | p-values |  | N | Means ± SE | R^2^ | p-values |
| pH | 210 | 7.88±0.09 | +0.15 | 0.03 |  | 230 | 7.95±0.1 | +0.16 | 0.02 |
| TDS (mg/l) | 96 | 249.11±34.33 | -0.02 | 0.87 |  | 99 | 185.94±17.44 | -0.08 | 0.45 |
| Conductivity (µs/cm) | 235 | 427.43±19.9 | +0.08 | 0.23 |  | 232 | 418.93±29.14 | +0.02 | 0.7 |
| Turbidity (FTU) | 235 | 209.37±26.17 | +0.21 | 0.001 |  | 220 | 285.7±109.28 | +0.34 | <0.001 |
| Ammonia | 71 | 0.53 ± 0.25 | +0.05 | 0.54 |  | 68 | 0.37 ± 0.11 | +0.022 | 0.8 |
| Phosphate | 92 | 0.41 ± 0.08 | -0.12 | 0.15 |  | 98 | 0.6 ± 0.13 | -0.056 | 0.54 |
| Nitrate | 101 | 1.71 ± 0.35 | -0.48 | <0.001 |  | 97 | 3.05 ± 0.61 | +0.07 | 0.41 |
| Calcium (mg/l) | 70 | 513.58±364.37 | -0.15 | 0.22 |  | 103 | 139.27±51.41 | -0.07 | 0.5 |
| Iron (mg/l) | 114 | 0.67±0.09 | -0.09 | 0.35 |  | 84 | 0.83±0.17 | -0.19 | 0.09 |
| Organophosphates (mg/l) | 134 | 7.94±0.46 | -0.06 | 0.46 |  | 126 | 15.04±4.67 | -0.27 | 0.002 |
| Aluminium (mg/l) | 90 | 0.76±0.26 | +0.05 | 0.61 |  | 110 | 0.56±0.13 | +0.21 | 0.03 |
| Sulphate (mg/l) | 124 | 92.71±5.49 | +0.07 | 0.44 |  | 91 | 81.82±4.58 | -0.27 | 0.009 |
| H_2_O_2_ (mg/l) | 104 | 3.78±1.04 | -0.09 | 0.36 |  | 84 | 9.48±2.39 | +0.28 | 0.01 |
| Temperature (°C) | 235 | 28.03±0.18 | +0.19 | 0.003 |  | 232 | 27.3±0.17 | +0.13 | 0.05 |

| **Intervention** | Non-intervention areas | | | |  | Intervention areas | | | |
| --- | --- | --- | --- | --- | --- | --- | --- | --- | --- |
| Parameters | N | Means ± SE | R^2^ | p-values |  | N | Means ± SE | R^2^ | p-values |
| pH | 51 | 7.45 ± 0.14 | +0.38 | 0.04 |  | 39 | 7.84 ± 0.2 | +0.09 | 0.56 |
| TDS (mg/l) | 51 | 627.28 ± 52.23 | -0.33 | 0.08 |  | 39 | 561.18 ± 72.59 | +0.22 | 0.18 |
| Conductivity (µs/cm) | 51 | -50.47 ± 24.38 | -0.1 | 0.62 |  | 39 | -56.13 ± 15.21 | +0.08 | 0.62 |
| Turbidity (FTU) | 64 | 255.42 ± 70.31 | +0.16 | 0.34 |  | 50 | 302.4 ± 156.21 | +0.28 | 0.06 |
| Ammonia (mg/l) | 64 | 3.54 ± 2.77 | -0.04 | 0.8 |  | 52 | 0.59 ± 0.15 | -0.035 | 0.81 |
| Nitrate (mg/l) | 64 | 1.92 ± 0.63 | +0.11 | 0.54 |  | 52 | 9.8 ± 6.16 | -0.21 | 0.14 |
| Phosphates (mg/l) | 64 | 0.4 ± 0.1 | -0.06 | 0.72 |  | 52 | 0.36 ± 0.06 | +0.02 | 0.87 |
| Organophosphates (mg/l) | 64 | 2.51 ± 1.16 | -0.33 | 0.05 |  | 52 | 2.47 ± 2.16 | +0.18 | 0.21 |
| Sulphate (mg/l) | 64 | 33.84 ± 5.65 | -0.51 | 0.001 |  | 52 | 30.73 ± 5.07 | +0.02 | 0.88 |
| H_2_O_2_ (mg/l) | 63 | 9.94 ± 1.66 | +0.45 | 0.008 |  | 52 | 8.71 ± 1.1 | +0.26 | 0.07 |
| Temperature (°C) | 51 | 28.49 ± 0.64 | -0.26 | 0.17 |  | 39 | 26.47 ± 0.54 | -0.006 | 0.97 |

N= number of aquatic habitats sampled and containing anopheline larvae; Mean = average concentration of the parameter in aquatic habitats with anopheline larvae ; SE: Standard error; R^2^= correlation coefficient between anopheline larval density and physico-chemical factor concentration, TDS: Total Dissolved Solids

H_2_O_2_: hydrogen peroxid

Supplementary Table S2: Effect of house characteristics on anophelines densities before and during intervention in non-intervention and intervention areas

|  | **Baseline** | | | | |  | **Intervention** | | | | | |
| --- | --- | --- | --- | --- | --- | --- | --- | --- | --- | --- | --- | --- |
|  | **Mean ± 95% CI** | |  | | |  | **Mean ± 95% CI** | |  | | | |
| **Characteristics** | **Non LCI** | **LCI** | **RR ± 95%CI** | **P value** | |  | **Non LCI** | **LCI** | **RR ± 95%CI** | **P value** | | |
| **Type of house** | | | | | | | | | | | | |
| Modern | 0.15± 0.01 | 0.22 ± 0.01 | 0.75 ± 0.12 | | 0.001 |  | 0.09 ± 0.008 | 0.04 ± 0.005 | 1.58 ± 0.38 | | < 0.001 | |
| Traditional | 0.27 ± 0.02 | 0.23 ± 0.02 | 1.22 ± 0.19 | | 0.006 |  | 0.11 ± 0.009 | 0.03 ± 0.005 | 2.98 ± 0.73 | | < 0.001 | |
| **Occupants per house** | | | | | | | | | | | | |
| > 5 | 0.20 ± 0.01 | 0.22 ± 0.01 | 0.97 ± 0.13 | | 0.62 |  | 0.09 ± 0.008 | 0.04 ± 0.005 | 1.88 ± 0.38 | | | < 0.001 |
| ≤ 5 | 0.23 ± 0.02 | 0.24 ± 0.03 | 1.01 ± 0.16 | | 0.95 |  | 0.12 ± 0.01 | 0.03 ± 0.005 | 2.66 ± 0.67 | | | < 0.001 |
| **Holes on walls** | |  |  | |  |  |  |  |  | | |  |
| No | 0.23 ± 0.01 | 0.20 ± 0.01 | 1.07 ± 013 | | 0.24 |  | 0.10 ± 0.008 | 0.029 ± 0.004 | 2.33 ± 0.48 | | | < 0.001 |
| Yes | 0.18 ± 0.01 | 0.28 ± 0.02 | 0.77 ± 0.14 | | 0.003 |  | 0.11 ± 0.01 | 0.058 ± 0.008 | 1.86 ± 0.47 | | | < 0.001 |
| **Eaves** | | | | | | | | | | | | |
| Closed | 0.20 ± 0.02 | 0.21 ± 0.01 | 0.96 ± 0.17 | | 0.59 |  | 0.09 ± 0.009 | 0.04 ± 0.005 | 1.74 ± 0.41 | | | < 0.001 |
| Opened | 0.22 ± 0.01 | 0.25 ± 0.01 | 0.94 ± 0.13 | | 0.36 |  | 0.11 ± 0.008 | 0.01 ± 0.006 | 2.40 0,52 | | | < 0.001 |
| **Ceiling** | |  |  | |  |  |  |  |  | | |  |
| No | 0.23 ± 0.01 | 0.29 ± 0.02 | 0.90 ± 0.12 | | 0.08 |  | 0.12 ± 0.008 | 0.04 ± 0.006 | 2.13 ± 0.41 | | | < 0.001 |
| Yes | 0.17 ± 0.01 | 0.16 ± 0.01 | 0.98 ± 0.19 | | 0.79 |  | 0.07 ± 0.009 | 0.03 ± 0.006 | 1.85 ± 0.52 | | | < 0.001 |
| **Screens on windows** | |  |  | |  |  |  |  |  | | |  |
| No | 0.21 ± 0.01 | 0.23 ± 0.01 | 0.96 ± 0.11 | | 0.48 |  | 0.11 ± 0.007 | 0.04 ± 0.004 | 2.15 ± 0.37 | | | < 0.001 |
| Yes | 0.20 ± 0.03 | 0.18 ± 0.02 | 1.06 ± 0.32 | | 0.67 |  | 0.10 ± 0.015 | 0.03 ± 0.007 | 2.17 ± 0.82 | | | < 0.001 |
| **Use of LLINs** | | | | | | | | | | | | |
| No | 0.15 ± 0.02 | 0.084 ± 0.01 | 1.17 ± 0.44 | | 0.35 |  | 0.05 ± 0.01 | 0.05 ± 0.015 | 1.22 ± 0.7 | | | 0.40 |
| Yes | 0.14 ± 0.01 | 0.08 ± 0.006 | 0.96 ± 0.11 | | 0.49 |  | 0.11 ± 0.006 | 0.04 ± 0.003 | 1.98 ± 0.33 | | | < 0.001 |
| **Vegetation close to the house** | | | | |  |  |  |  |  | | |  |
| No | 0.19 ± 0.02 | 0.26 ± 0.02 | 0.91 ± 0.24 | | 0.43 |  | 0.09 ± 0.01 | 0.04 ± 0.007 | 2.38 ± 0.82 | | | < 0.001 |
| Yes | 0.21 ± 0.01 | 0.22 ± 0.01 | 1.01 ± 0.11 | | 0.90 |  | 0.11 ± 0.007 | 0.04 ± 0.004 | 2.17 ± 0.39 | | | < 0.001 |
| **Aquatic habitats less than 10m from the house** | | | | |  |  |  |  |  | | |  |
| No | 0.19 ± 0.02 | 0.22 ± 0.02 | 0.78 ± 0.2 | | 0.04 |  | 0.08 ± 0.01 | 0.03 ± 0.008 | 2.09 ± 0.84 | | | < 0.001 |
| Yes | 0.22 ± 0.01 | 0.20 ± 0.01 | 1.03 ± 0.12 | | 0.59 |  | 0.11 ± 0.002 | 0.04 ± 0.004 | 2.27 ± 0.39 | | | < 0.001 |
| **Number of Bedrooms** |  |  |  | |  |  |  |  |  | | |  |
| ˂ 5 | 0.22 ± 0.01 | 0.22 ± 0.01 | 0.99 ± 0.12 | | 0.97 |  | 0.10 ± 0.008 | 0.04 ± 0.005 | 1.86 ± 0.36 | | | < 0.001 |
| ≥ 5 | 0.16 ± 0.03 | 0.14 ± 0.031 | 1.02 ± 0.45 | | 0.92 |  | 0.07 ± 0.01 | 0.003 ± 0.04 | 2.33 ± 1.76 | | | 0.003 |
| **Number of Windows** |  |  |  | |  |  |  |  |  | | |  |
| ˂ 5 | 0.22 ± 0.01 | 0.21 ± 0.01 | 1.09 ± 0.14 | | 0.19 |  | 0.11 ± 0.008 | 0.04 ± 0.004 | 2.22 ± 0.42 | | | 0.001 |
| ≥ 5 | 0.18 ± 0.02 | 0.25 ± 0.02 | 0.84 ± 0.16 | | 0.05 |  | 0.11 ± 0.01 | 0.04 ± 0.007 | 2.08 ± 0.7 | | | 0.001 |
| **Number of Doors** |  |  |  | |  |  |  |  |  | | |  |
| ˂ 5 | 0.22 ± 0.01 | 0.21 ± 0.01 | 1.03 ± 0.11 | | 0.57 |  | 0.11 ± 0.007 | 0.04 ± 0.004 | 2.25 ± 0.35 | | | 0.001 |
| ≥ 5 | 0.12 ± 0.02 | 0.13 ± 0.14 | 0.62 ± 0.86 | | 0.28 |  | 0.06 ± 0.02 | 0.05 ± 0.02 | 1.52 ± 1.45 | | | 0.22 |

Mean = average number of mosquitoes collected per trap per night

Non larviciding intervention area (Non LCI), Larviciding Intervention area (LCI), RR = Relative Risk of being exposed to anopheline bites between non-intervention and intervention areas for each parameter before and during the intervention

95% CI = 95% Confidence Interval

LLINs=Long Lasting Insecticidal Nets

Supplementary Table S3: Influence of larviciding treatments on non-target organisms

| **Groups** | **Species** | **Non-intervention areas** (n=145) | **intervention areas** (n=145) |
| --- | --- | --- | --- |
| **Copepods** | *Cyclopidae* spp. | +++ | ++ |
|  | *Copepodes* spp. | +++ | +++ |
|  | *Calanoide* spp. | +++ | + |
| **Rotifers** | *Rotaria rotatoria* | ++++ | ++ |
|  | *Brachionus patulus patulus* | ++++ | ++ |
|  | *Notholca salina* | + | - |
|  | *Notholca striava* | - | - |
|  | *Kurzia media* | + | + |
|  | *Lepadella quadricarinata* | + | ++ |
|  | *Lophocharis salpina* | - | - |
|  | *Lecane physalis* | - | +/- |
|  | *Platyias quadricormis* | - | + |
|  | *Keratela* spp. | - | + |
|  | *Lecane clara* | - | +/- |
|  | *Brachionus bidentata* |  | +/- |
|  | *Brachionus ferficala* | - | + |
|  | *Frola zaralli* | - | + |
|  | *Brachionus budapestinensis* | + | - |
|  | *Trichocerca diurella* | - | ++ |
|  | *Colurella geophila geophila* | +/- | ++ |
| **Cladocerans** | *Alona protzi* | - | +/- |
|  | *Alona weltneri* | +/- | +/- |
|  | *Alona guttata* | - | +/- |
|  | *Alona quadrangularis* | - | +/- |
|  | *Alonella exugua* | - | + |
|  | *Daphnia similis* | - | + |
|  | *Pleuroxus inermis* | - | +/- |
|  | *Acroperus harpae* | - | + |
|  | *Simocephalus exspinosus* | - | +/- |
|  | *Oxyurella terulcaudia* | - | + |
|  | *Sida crystallina* | - | +/- |
|  | *Chydorus ovalis* | - | +/- |
|  | *Disparalona rostrata* | - | +/- |
|  | *Chydorus piger* | - | + |
|  | *Oxyurella terulcaudia* | - | + |
|  | *Alona rectangula* | - | + |
|  | *Diaphranosoma brachyunum* | ++ | +/- |
|  | *Blapertura affinis* | +++ | - |
|  | *Camphocercus rectirostris* | - | + |
|  | *Ceriodaphnia rotunda* | - | + |
|  | *Moina micrura* | ++++ | ++++ |
|  | *Moina macrocopa* | ++++ | ++++ |
|  | Cyprus spp. | ++ | + |
| **Ostracods** | *Ostracod* spp. | +/- | - |

Absent = - ; x < 1%  = +/- ; 1% < x < 5% = + ; 5% < x < 25% = ++ ; 25% < x < 50% = +++ ; x> 50% ++++ ; x = density ; n= number of prospected aquatic habitats
